# Low expression of ZSCAN4 predicts unfavorable outcome in urothelial carcinoma of upper urinary tract and urinary bladder

**DOI:** 10.1186/s12957-023-02948-4

**Published:** 2023-02-25

**Authors:** Hong-Lin He, Hong-Yue Lai, Ti-Chun Chan, Chung-Hsi Hsing, Steven K. Huang, Kun-Lin Hsieh, Tzu-Ju Chen, Wan-Shan Li, Yu-Hsuan Kuo, Yow-Ling Shiue, Chien-Feng Li

**Affiliations:** 1 Department of Pathology, E-DA Cancer Hospital, I-Shou University, Kaohsiung, Taiwan; 2 Department of Medical Research, Chi Mei Medical Center, Tainan, Taiwan; 3 National Institute of Cancer Research, National Health Research Institutes, Tainan, Taiwan; 4 Department of Anesthesiology, Chi Mei Medical Center, Tainan, Taiwan; 5 Division of Urology, Department of Surgery, Chi Mei Medical Center, Tainan, Taiwan; 6 Department of Biotechnology, Chia Nan University of Pharmacy and Science, Tainan, Taiwan; 7 Department of Clinical Pathology, Chi Mei Medical Center, Tainan, Taiwan; 8 Department of Medical Technology, Chung Hwa University of Medical Technology, Tainan, Taiwan; 9 Department of Pathology, Chi Mei Medical Center, Tainan, Taiwan; 10 Division of Hematology and Oncology, Department of Internal Medicine, Chi-Mei Medical Center, Tainan, Taiwan; 11 College of Pharmacy and Science, Chia Nan University, Tainan, Taiwan; 12 Institute of Precision Medicine, National Sun Yat-Sen University, Kaohsiung, Taiwan

**Keywords:** ZSCAN4, Urothelial carcinoma, UC, Prognosis, Tumor suppressor

## Abstract

**Background:**

With the advance in genome-wide analyses, genetic alternations have been found to play an important role in carcinogenesis and aggressiveness of UC. Through bioinformatic analysis of gene expression profiles of urinary bladder urothelial carcinoma (UBUC) from publicly available GEO dataset (GSE31684), *Zinc finger and SCAN domain containing 4 (ZSCAN4)* was identified as a significant downregulated gene in muscle-invasive bladder cancer when compared with non-muscle-invasive bladder cancer.

**Methods:**

The expression of ZSCAN4 was evaluated by immunohistochemistry in 340 upper urinary tract urothelial carcinomas (UTUCs) and 295 UBUCs. The expression profiles of ZSCAN4 and potential signaling pathways were analyzed bioinformatically.

**Results:**

In UTUC, low expression of ZSCAN4 was significantly associated with advanced primary pT stage (*P* = 0.011), increased nodal metastasis (*P* = 0.002) and increased vascular invasion (*P* = 0.019). In UBUC, low expression of ZSCAN4 was significantly correlated with advanced primary pT stage (*P* < 0.001), increased nodal metastasis (*P* = 0.001), high histological grade (*P* = 0.003) and increased vascular invasion (*P* = 0.003). In survival analysis, low expression of ZSCAN4 acted as an independent negative prognostic factor for disease-specific survival and metastasis-free survival both in UTUC and UBUC. Gene ontology analysis showed that *ZSCAN4* mRNA and its co-downregulated genes are associated with the mitotic cell cycle.

**Conclusions:**

Low expression of ZSCAN4 predicted worse outcome in urothelial carcinoma and might have potential regulatory role in cell mitosis.

**Supplementary Information:**

The online version contains supplementary material available at 10.1186/s12957-023-02948-4.

## Introduction

Urothelial carcinoma (UC) is the most common epithelial malignancy involving the urinary system. Some environmental factors contribute to increasing risk of UC, including tobacco smoking, intake of arsenic-contaminated water, occupational exposure to aromatic amines and polycyclic hydrocarbons, exposure to ionizing radiation and chronic infection of Schistosoma species [1]. Recent genome-wide studies suggested that molecular alternations play an important role in carcinogenesis and aggressiveness of UC. Through analysis of the mRNA expression profiles, multiple molecular subtypes are identified according to their different expression levels of certain key prognostic markers, such as fibroblast growth factor receptor 3 (FGFR3), GATA binding protein 3 (GATA3), forkhead box A1 (FOXA1), uroplakin 3A (UPK3A), and erb-b2 receptor tyrosine kinase 2 (ERBB2) [2–5]. Molecular stratification may provide better diagnostic, prognostic and/or predictive value than conventional pathologic classification. The diagnostic and prognostic data are often associated with histological grading and classification while the predictive data are linked with the therapeutic response. Moreover, insights into the molecular basis of human cancer provide information of biological functions of neoplasms.

Deletion in chromosome 9 are the earliest genetic events that occurs in the divergent pathways of tumorigenesis in bladder cancer, which leads to two distinct phenotypes: non-muscle-invasive and muscle-invasive urothelial carcinomas. Candidate tumor suppressor genes affected by chromosome 9 deletion includes cyclin-dependent kinase inhibitor 2A (*CDKN2A*) and cyclin-dependent kinase inhibitor 2B (*CDKN2B*) at 9p21 [6, 7], patched 1 (*PTCH1*) at 9q22 [8, 9], deleted in bladder cancer 1 (*DBC1*) at 9q32–33 [10], and tuberous sclerosis 1 (*TSC1*) at 9q34 [11]. The main genetic alterations in non-muscle-invasive urothelial carcinoma involves three receptor tyrosine kinase genes, *FGFR3*, v‑Ha-ras Harvey rat sarcoma viral oncogene homolog (*HRAS*), and phosphatidylinositol-4,5-bisphosphate 3-kinase catalytic subunit alpha (*PIK3CA*) [12–14]. By contrast, alterations involved in *P53* and RB transcriptional corepressor 1 (*RB1*) lead to progression to non-invasive high-grade and muscle-invasive urothelial carcinomas [15–17].

To identify potential candidate genes associated with aggressiveness of UC, we analyzed gene expression profiles of urinary bladder urothelial carcinomas (UBUCs) from publicly available Gene Expression Omnibus (GEO) dataset with the accession number of GSE31684 [18]. The analytic data suggested that *Zinc finger and SCAN domain containing 4 (ZSCAN4)* was found to be significantly associated with tumor invasion depth, characterized by significant downregulation in muscle-invasive UBUCs (T2–T4) when compared with non-muscle-invasive UBUCs (Ta-T1). Its strong statistically significance (*P* < 0.0001) draws our attention to select ZSCAN4 for further study. In this study, we tried to validate the prognostic significance of ZSCAN4 in UC patients and to investigate its potential regulatory signaling pathways.

## Materials and methods

### Data mining of publicly available transcriptome

We performed data mining of publicly available transcriptome of urinary bladder urothelial carcinoma with the accession number of GSE31684 (https://www.ncbi.nlm.nih.gov/geo/query/acc.cgi?acc=GSE31684) in Gene Expression Omnibus (GEO) database, which includes 93 UBUCs. The raw CEL files were analyzed on Affymetrix Human Genome U133 Plus 2.0 Array platform by using the Nexus Expression 3 software (BioDiscovery, EI Segundo, CA, USA). All probes were included in the analysis. Supervised comparative analyses were performed to identify potential genes that were differentially expressed between muscle-invasive (T2–T4) and non-muscle-invasive (Ta-T1) bladder cancer. Genes were selected based on the condition that a *P* value is less than 0.01 and log2 fold gene expression change more than ± 0.1. Further survival analysis was performed to evaluate the prognostic significance of this gene.

### Patients and tumor samples

Tumor tissues with available paraffin-embedded tissue blocks were obtained from the archives of Chi-Mei medical center for tissue microarray construction, including 340 upper urinary tract urothelial carcinomas (UTUCs) and 295 UBUCs. Those with squamous, glandular or neuroendocrine component were excluded. The acquisition of clinical samples was approved by the Institutional Review Board (IRB10302015) of Chi-Mei medical center. Patients’ characteristics were described previously [19]. Criteria of histopathological diagnosis and assessment for various histopathological parameters were based on the updated 4th edition of WHO classification of the Urinary System and Male Genital Organs.

### Immunohistochemistry and scoring

The immunohistochemical staining was performed on 4-μm-thick sections from formalin fixed paraffin embedded tissue blocks according to the manufacturer’s recommendations. After antigen retrieval, the slides were incubated with a primary antibody against ZSCAN4 (Abcam, ab106646, 1:50). The assessment of ZSCAN4 staining was based on H-score method. The H-score was calculated according to the following formula: 3 × strongly positive tumor cells (%) + 2 × moderately positive tumor cells (%) + 1 × weakly positive tumor cells (%). Tumors with high and low expression of ZSCAN4 are defined by their H-scores that are higher and lower than the median, respectively.

### Functional annotation of The Cancer Genome Atlas (TCGA) data

To correlate *ZSCAN4* with unrealized functions in UC, the associations between the levels of *ZSCAN4* mRNA and its co-expressed genes in the bladder urothelial carcinoma dataset (*n* = 411) from The Cancer Genome Atlas (TCGA) database were analyzed using the cBioPortal online platform (http://cbioportal.org). The top 200 transcripts with either positive associations or negative associations with *ZSCAN4* were further explored using *the Gene Ontology* (*GO*) classification system (http://geneontology.org/) according to cellular components, molecular functions, or biological processes and were graded by *fold enrichment* for functional annotation. An R script with ggplot2 package was used to present representative GO terms.

### Statistical analyses

All analyses were performed using SPSS Version 20.0 software (Armonk, NY: IBM Corp., USA). For associations between immunohistochemical expression of ZSCAN4 and clinicopathological parameters, we used Pearson’s chi-squared test to identify significant differences between variables. Kaplan–Meier plots were applied to evaluate survival data, including disease-specific survival (DSS) and metastasis-free survival (MeFS). The prognostic significances of each parameter with suitable cut-offs were determined by the log-rank test. The Cox proportional hazards regression model was used to measure the effects of variables on survival rates. The level of significance was determined according to two-sided tests with a cut-off *P* value of 0.05.

## Results

### *ZSCAN4* is identified as a significant downregulated gene in muscle-invasive UBUCs (T2-T4) when compared with non-muscle-invasive UBUCs (Ta-T1)

**Fig. 1:**
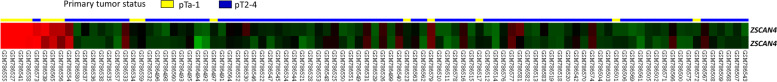
Bioinformatic analysis of gene expression profiles in urinary bladder urothelial carcinoma (GEO database: GSE31684). Downregulation of ZSCAN4 was found in muscle invasive bladder cancer when compared with non-muscle-invasive bladder cancer

Though analysis of publicly available transcriptome of UBUC (GSE31684), *ZSCAN4* was found to be the most significantly downregulated in muscle-invasive UBUCs (T2–T4) when compared with non-muscle-invasive UBUCs (Ta–T1), displaying significant downregulated fold change (Log_2_ ratio at − 0.9972 and − 0.7781, both *P* < 0.0001, Fig. 1 and Table 1).

**Table 1:** Exploration of ZSCAN4 alteration during the progression of urothelial carcinoma of urinary bladder (GSE31684)

Probe	Comparing T2–4 to Ta–T1		Gene Symbol	Biological process	Molecular function
	Log2 ratio	*P* value			
1552851_at	− 0.9972	*P* < 0.0001	*ZSCAN4*	Regulation of transcription; DNA-dependent, transcription	DNA binding, metal ion binding, nucleic acid binding, transcription factor activity, zinc ion binding
1552852_a_at	− 0.7781	*P* < 0.0001	*ZSCAN4*	Regulation of transcription; DNA-dependent, transcription	DNA binding, metal ion binding, nucleic acid binding, transcription factor activity, zinc ion binding

### Low mRNA transcript level of ZSCAN4 predicts worse outcome in the UBUC transcriptome (GSE31684)

**Fig. 2:**
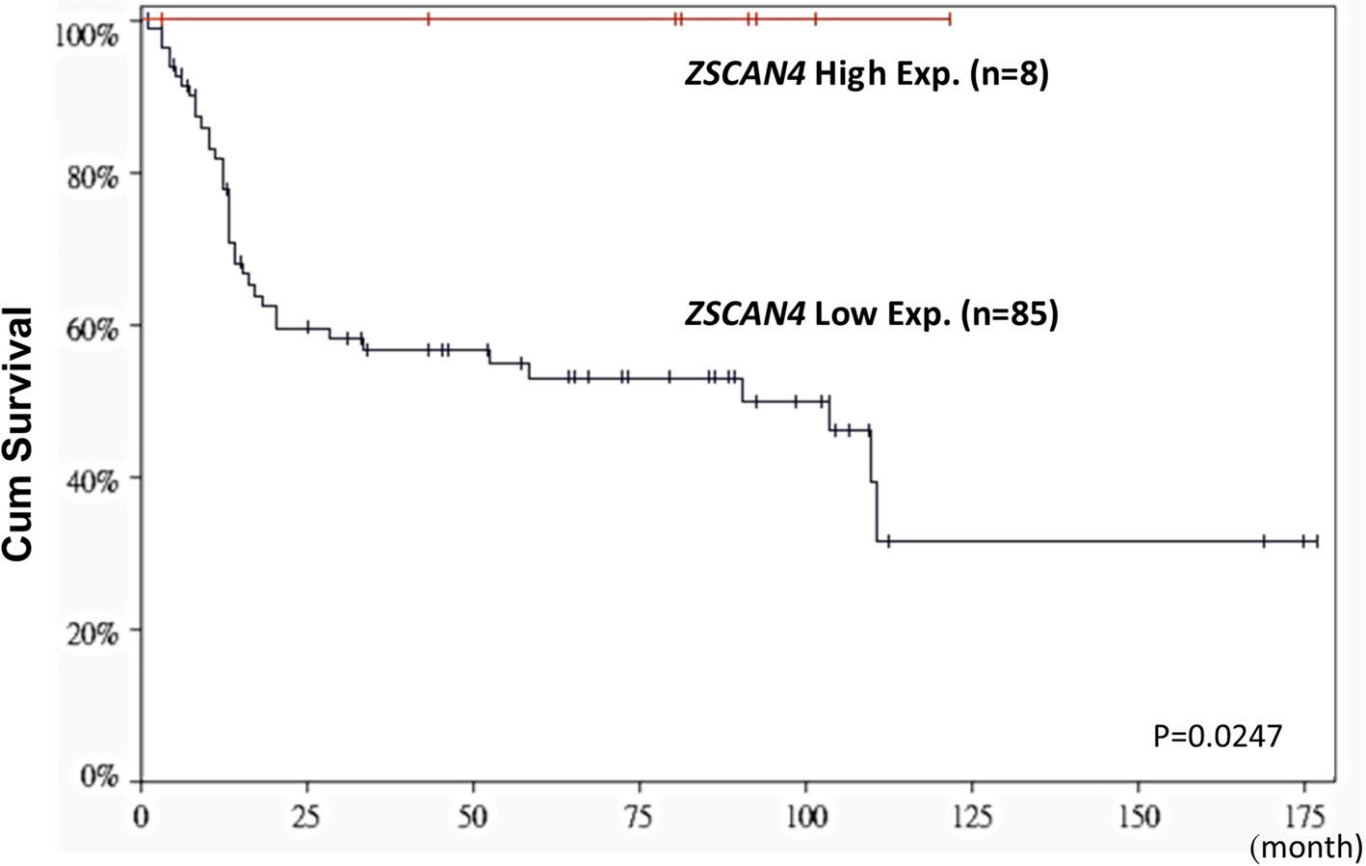
Kaplan–Meier plot of GSE31684 demonstrated that low *ZSCAN4* mRNA level predicts worse overall survival (*P* = 0.0247)

To further investigate the prognostic significance of ZSCAN4 in UBUC, we performed survival analysis for the UBUC transcriptome (GSE31684), consisting of 93 cases. Among them, 8 cases had high mRNA expression levels of *ZSCAN4* while the other 85 cases had low expression. Of note, low expression of ZSCAN4 was significantly associated with worse overall survival (Fig. 2).

### Low protein expression of ZSCAN4 is associated with advanced disease status in UTUC and UBUC

**Fig. 3:**
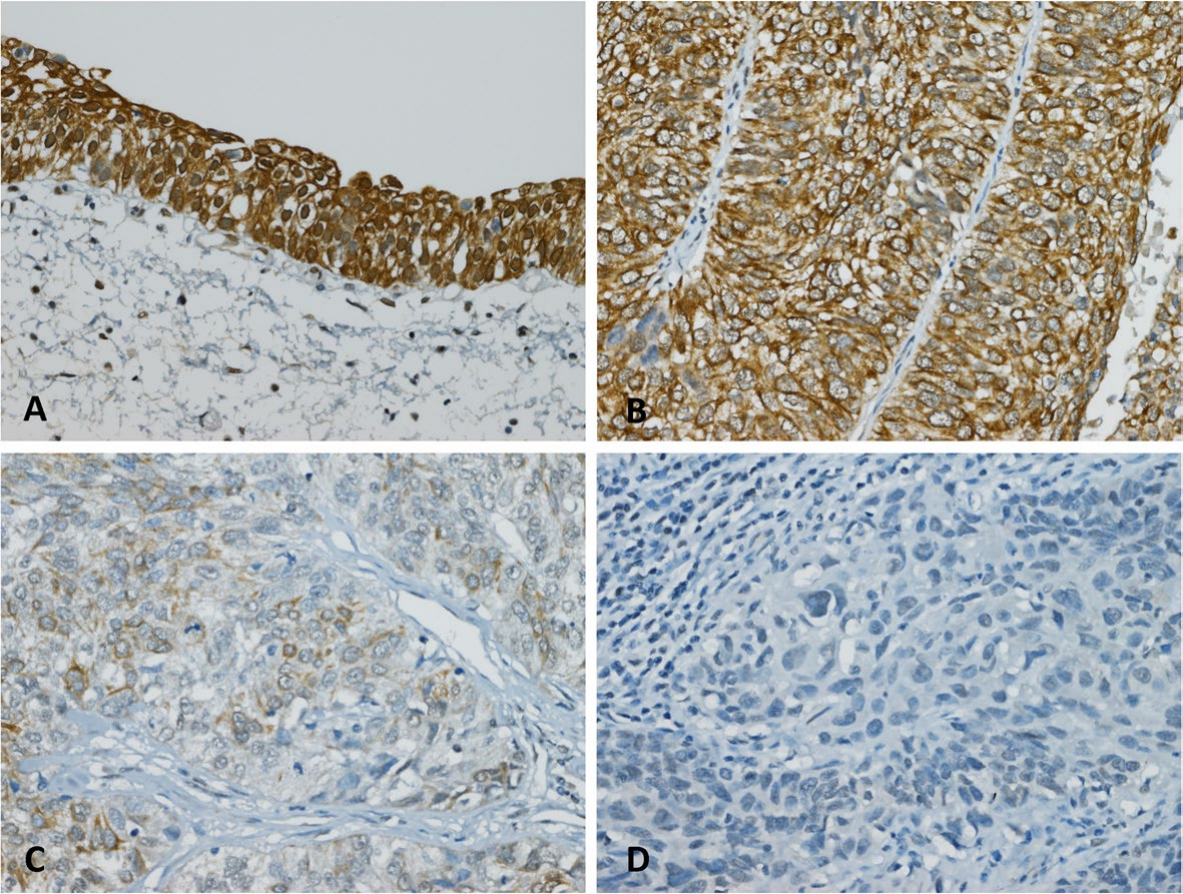
Immunohistochemical staining of ZSCAN4 in representative cases. The expression intensities of ZSCAN4 immunostains were strong in normal urothelium (**A**) and non-invasive urothelial carcinoma (**B**), weak in superficially invasive urothelial carcinoma (**C**), and faint or absent in muscle-invasive urothelial carcinoma (**D**)

The immunoexpression of ZSCAN4 was successfully evaluated with H-score method in all UC tissue samples (Fig. 3). As shown in Table 2, the association between ZSCAN4 expression levels and various clinicopathological parameters were statistically analyzed. In UTUC and UBUC, there was no significant difference in gender, age, perineural invasion or mitotic rate according to the expression status of ZSCAN4. In UTUC, low expression of ZSCAN4 was significantly associated with advanced primary pT stage (*P* = 0.011), increased nodal metastasis (*P* = 0.002) and increased vascular invasion (*P* = 0.019). In UBUC, low expression of ZSCAN4 was significantly correlated with advanced primary pT stage (*P* < 0.001), increased nodal metastasis (*P* = 0.001), high histological grade (*P* = 0.003) and increased vascular invasion (*P* = 0.003). These findings indicated that there is a close correlation between low ZSCAN4 expression and aggressive tumor behavior in patients with UTUC or UBUC.

**Table 2:** Associations of ZSCAN4 expression with clinicopathological features in urothelial carcinoma

Parameter	Category	Upper urinary tract urothelial carcinoma				Urinary bladder urothelial carcinoma			
		Case no	ZSCAN4 expression		*p* value	Case no	ZSCAN4 expression		*p* value
			High	Low			High	Low	
Gender	Male	158	85	73	0.192	216	114	102	0.094
	Female	182	85	97		79	33	46	
Age (years)	< 65	138	64	74	0.269	121	60	61	0.944
	≥ 65	202	106	96		174	87	87	
Tumor site	Renal pelvis	141	66	75	0.011*	–	–	–	–
	Ureter	150	87	63		–	–	–	–
	Renal pelvis and ureter	49	17	32		–	–	–	–
Multifocality	Single	278	146	132	0.049*	–	–	–	–
	Multifocal	62	24	38		–	–	–	–
Primary tumor (T)	Ta	89	55	34	0.011*	84	57	27	< 0.001*
	T1	92	48	44		88	47	41	
	T2–T4	159	67	92		123	43	80	
Nodal status (N)	Negative (N0)	312	164	148	0.002*	266	141	125	0.001*
	Positive (N1–N2)	28	6	22		29	6	23	
Histological grade	Low grade	56	26	30	0.559	56	38	18	0.003*
	High grade	284	144	140		239	109	130	
Vascular invasion	Absent	234	127	107	0.019*	246	132	114	0.003*
	Present	106	43	63		49	15	34	
Perineural invasion	Absent	321	161	160	0.813	275	139	136	0.362
	Present	19	9	10		20	8	12	
Mitotic rate (per 10 high power fields)	< 10	173	92	81	0.233	139	72	67	0.523
	> = 10	167	78	89		156	75	81	

^*^Statistically significant

### Low protein expression of ZSCAN4 predicts worse outcome in UTUC and UBUC

**Fig. 4:**
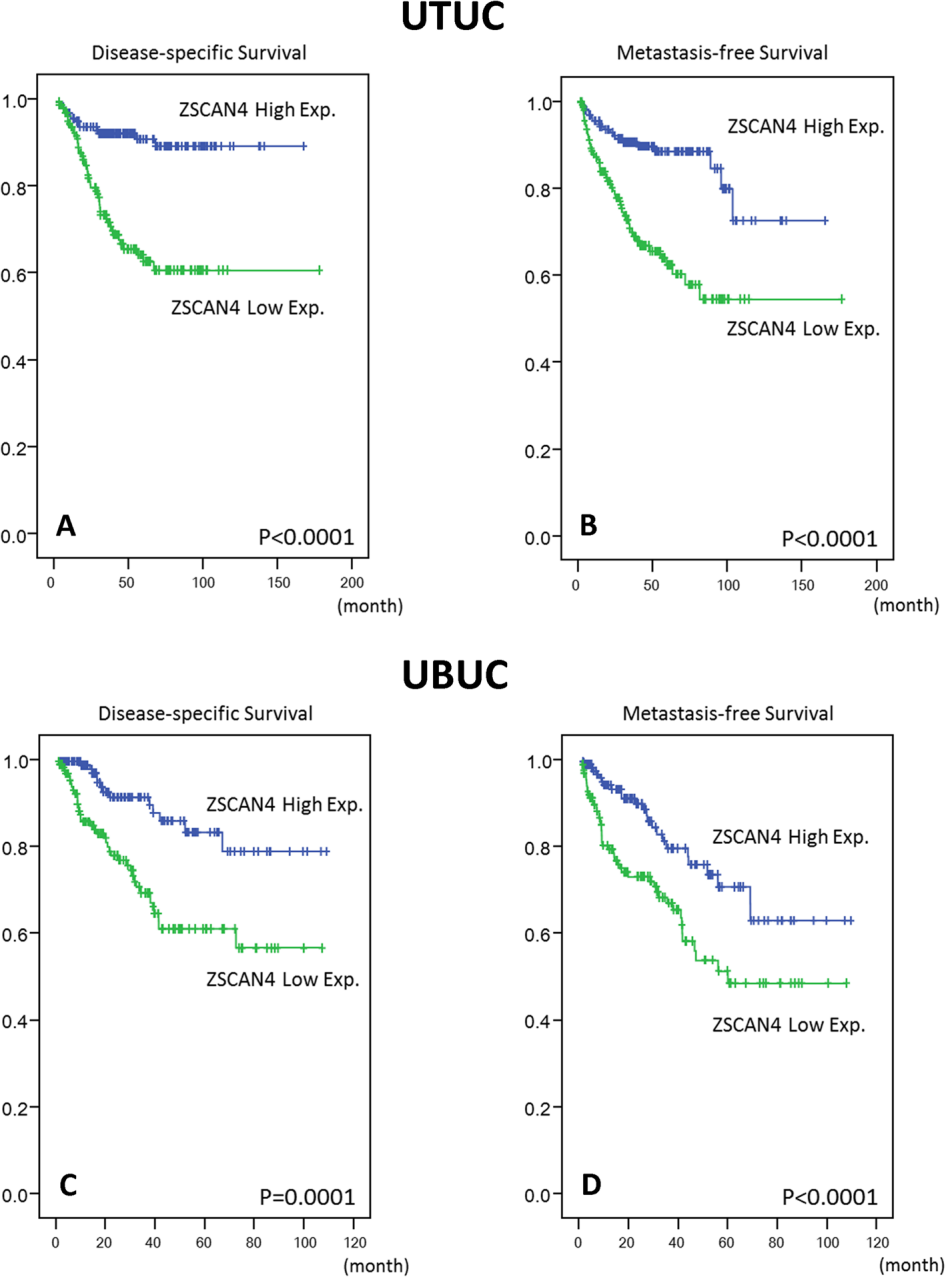
Kaplan–Meier analyses of disease-specific survival (DSS) and metastasis-free survival (MeFS) according to ZSCAN4 immunoexpression status. Low expression of ZSCAN4 was significantly associated with worse DSS and MeFS both in UTUC (**A** and **B**) and UBUC (**C** and **D**)

The results of univariate log-rank analyses and multivariate analyses that investigate the impact of ZSCAN4 expression and various clinicopathological variables on survival in patients with UTUC and UBUC are shown in Tables 3 and 4, respectively. In patients with UTUC (Table 3), low expression of ZSCAN4 predicted worse DSS (*P* < 0.0001) (Fig. 4A) and MeFS (*P* < 0.0001) (Fig. 4B). In addition, tumor location, multifocality, advanced primary pT stage, presence of nodal metastasis, high histologic grade, increased vascular invasion, and increased perineural invasion was significantly associated with worse DSS and/or MeFS. At multivariate analyses, low expression of ZSCAN4 remain acted as an independent negative prognostic factor for DSS (95% CI 1.572–5.667, *P* = 0.001) and MeFS (95% CI 1.498–4.548, *P* = 0.001), along with multifocality (*P* = 0.010 in DSS; *P* = 0.010 in MeFS), advanced pT stage (*P* = 0.043 in DSS), presence of nodal metastasis (*P* < 0.001 in DSS; *P* = 0.009 in MeFS), high histologic grade (*P* = 0.007 in DSS; *P* = 0.007 in MeFS), increased vascular invasion (*P* = 0.004 in MeFS), and increased perineural invasion (*P* < 0.001 in DSS; *P* = 0.003 in MeFS). In UBUC patients (Table 4), low expression of ZSCAN4 was also significantly associated with worse DSS (*P* = 0.0001) (Fig. 4C) and MeFS (*P* < 0.0001) (Fig. 4D). Moreover, advanced primary pT stage, presence of nodal metastasis, high histologic grade, increased vascular invasion, increased perineural invasion, and high mitotic rate were significantly predicted worse DSS and/or MeFS. At multivariate analyses, low expression of ZSCAN4 still emerged as an independent negative prognostic factor for DSS (95% CI 1.382–5.123, *P* = 0.003) and MeFS (95% CI 1.010–2.759, *P* = 0.046), along with advanced pT stage (*P* < 0.001 in DSS; *P* = 0.002 in MeFS), increased perineural invasion (*P* = 0.023 in DSS), and increased mitotic rate (*P* = 0.003 in DSS; *P* = 0.006 in MeFS). These data suggested that low ZSCAN4 expression significantly predicted worse clinical outcome in patients with UTUC or UBUC.

**Table 3:** Effects of ZSCAN4 expression and clinicopathological parameters on prognosis in upper urinary tract urothelial carcinoma

Parameter	Category	Case no	Disease-specific survival					Metastasis-free survival				
			Univariate analysis		Multivariate analysis			Univariate analysis		Multivariate analysis		
			No. of event	*p* value	R.R	95% C.I	*p* value	No. of event	*p* value	R.R	95% C.I	*p* value
Gender	Male	158	28	0.8286	–	–	–	32	0.7904	–	–	–
	Female	182	33		–	–	–	38		–	–	–
Age (years)	< 65	138	26	0.9943	–	–	–	30	0.8470	–	–	–
	≥ 65	202	35		–	–	–	40		–	–	–
Tumor laterality	Right	177	34	0.7366	–	–	–	38	0.3074	–	–	–
	Left	154	26		–	–	–	32		–	–	–
	Bilateral	9	1		–	–	–	0		–	–	–
Tumor site	Renal pelvis	141	24	0.0079*	1	–	0.978	31	0.0659	–	–	–
	Ureter	150	22		0.990	0.268–3.661		25		–	–	–
	Renal pelvis and ureter	49	15		0.925	0.222–3.851		14		–	–	–
Multifocality	Single	273	48	0.0026*	1	–	0.010*	52	0.0127*	1	–	0.010*
	Multifocal	62	18		2.981	1.305–6.807		18		2.610	1.201–3.732	
Primary tumor (T)	Ta	89	2	< 0.0001*	1	–	0.043*	4	< 0.0001*	1	–	0.301
	T1	92	9		2.938	0.620–13.913		15		2.249	0.727–6.960	
	T2–T4	159	50		5.736	1.273–26.099		51		2.467	0.767–7.914	
Nodal status (N)	Negative (N0)	312	42	< 0.0001*	1	–	< 0.001*	55	< 0.0001*	1	–	0.009*
	Positive (N1–N2)	28	19		4.240	2.233–8.052		15		2.320	1.228–4.380	
Histological grade	Low grade	56	4	0.0215*	1	–	0.007*	3	0.0027*	1	–	0.007*
	High grade	284	57		4.743	1.539–14.619		67		5.232	1.557–17.582	
Vascular invasion	Absent	234	24	< 0.0001*	1	–	0.257	26	< 0.0001*	1	–	0.004*
	Present	106	37		1.419	0.774–2.600		44		2.468	1.327–4.591	
Perineural invasion	Absent	321	50	< 0.0001*	1	–	< 0.001*	61	< 0.0001*	1	–	0.003*
	Present	19	11		4.183	1.977–8.851		9		3.184	1.485–6.825	
Mitotic rate (per 10 high power fields)	< 10	173	27	0.167	–	–	–	30	0.0823	–	–	–
	> = 10	167	34		–	–	–	40		–	–	–
ZSCAN4 expression	High	170	13	< 0.0001*	1	–	0.001*	19	< 0.0001*	1	–	0.001*
	Low	170	48		2.984	1.572–5.667		51		2.610	1.498–4.548	

^*^Statistically significant

**Table 4:** Effects of ZSCAN4 expression and clinicopathological parameters on prognosis in urinary bladder urothelial carcinoma

Parameter	Category	Case no	Disease-specific survival					Metastasis-free survival				
			Univariate analysis		Multivariate analysis			Univariate analysis		Multivariate analysis		
			No. of event	*p* value	R.R	95% C.I	*p* value	No. of event	*p* value	R.R	95% C.I	*p* value
Gender	Male	216	41	0.4446	–	–	–	60	0.2720	–	–	–
	Female	79	11		–	–	–	16		–	–	–
Age (years)	< 65	121	17	0.1136	–	–	–	31	0.6875	–	–	–
	≥ 65	174	35		–	–	–	45		–	–	–
Primary tumor (T)	Ta	84	1	< 0.0001*	1	–	< 0.001*	4	< 0.0001*	1	–	0.002*
	T1	88	9		6.413	0.678–60.682		23		5.222	1.508–18.089	
	T2–T4	123	42		25.816	2.794–238.553		49		7.591	2.179–26.449	
Nodal status (N)	Negative (N0)	266	41	0.0002*	1	–	0.827	61	< 0.0001*	1	–	0.103
	Positive (N1–N2)	29	11		1.083	0.530–2.215		15		1.674	0.901–3.109	
Histological grade	Low grade	56	2	0.0013*	1	–	0.823	5	0.0007*	1	–	0.803
	High grade	239	50		0.836	0.174–4.021		71		1.143	0.488–1.592	
Vascular invasion	Absent	246	37	0.0024*	1	–	0.089	54	0.0001*	1	–	0.676
	Present	49	15		0.553	0.279–1.095		22		0.882	0.601–1.929	
Perineural invasion	Absent	275	44	0.0001*	1	–	0.023*	66	0.0007*	1	–	0.103
	Present	20	8		2.630	1.142–6.058		10		1.854	0.882–3.899	
Mitotic rate (per 10 high power fields)	< 10	139	12	< 0.0001*	1	–	0.003*	23	< 0.0001*	1	–	0.006*
	> = 10	156	40		2.734	1.401–5.335		53		2.051	1.223–3.438	
ZSCAN4 expression	High	147	13	0.0001*	1	–	0.003*	26	< 0.0001*	1	–	0.046*
	Low	148	39		2.661	1.382–5.123		50		1.669	1.010–2.759	

^*^Statistically significant

### ZSCAN4 downregulation may be linked to high mitotic activity

**Fig. 5:**
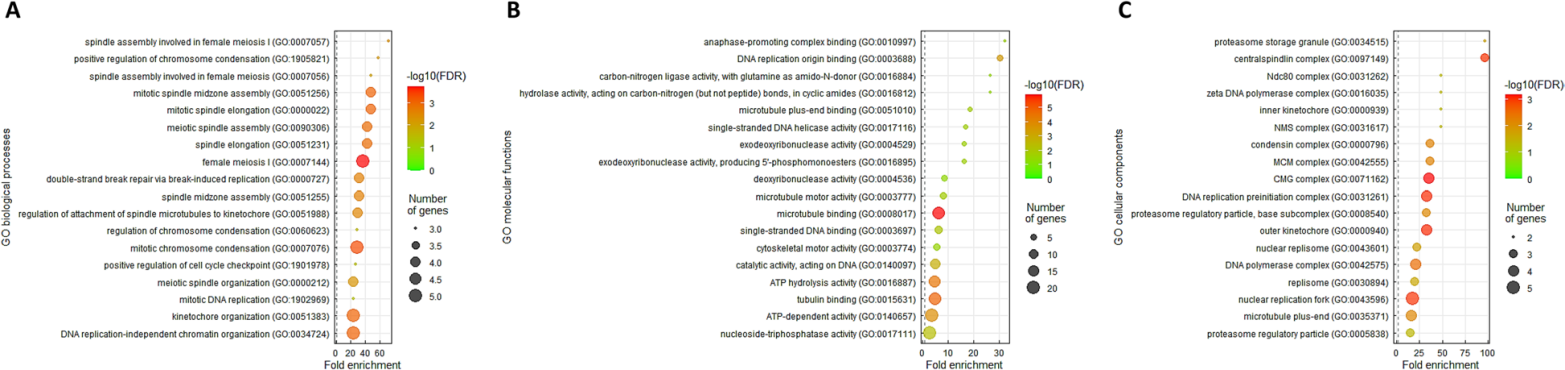
The significant GO terms enriched in *ZSCAN4* upregulation. The top 200 transcripts with negative associations with *ZSCAN4* were explored using the GO classification system according to **A** biological processes, **B** molecular functions, or **C** cellular components and were graded by fold enrichment for functional annotation. An R script with ggplot2 package was used to present representative GO terms

A gene co-expression assessment was performed to correlate *ZSCAN4* with unrealized functions in UC. Using the BLCA dataset (*n* = 411) from the TCGA database, we examined the top 200 transcripts that were positively associated (Supplementary Table S1) or negatively associated (Supplementary Table S2) with *ZSCAN4*. Next, these genes were functionally annotated by means of the GO classification system. In the context of biological processes (Fig. 5A), the top terms negatively associated with *ZSCAN4* comprised spindle assembly involved in female meiosis I (GO 0,007,057, fold enrichment 72.86), positive regulation of chromosome condensation (GO 1,905,821, fold enrichment 58.29), and mitotic spindle elongation (GO 0,000,022, fold enrichment 48.57). In terms of molecular functions (Fig. 5B) and cellular components (Fig. 5C), the most significant terms negatively associated with *ZSCAN4* were anaphase-promoting complex binding (GO 0,010,997, fold enrichment: 32.38) and centralspindlin complex (GO: 0,097,149, fold enrichment 97.15), respectively. As the mitotic rate has been used to measure how fast cancer cells are dividing (proliferating) and growing, tumors mostly have higher mitotic activity than normal tissues. Accordingly, our observations disclosed that the levels of *ZSCAN4* mRNA and its co-downregulated genes are greatly associated with the mitotic cell cycle, suggesting that ZSCAN4 is more likely to play a role in the suppression of UC progression.

## Discussion

In this study, we found that low expression of ZSCAN4 was significantly associated with advanced disease status and key pathological parameters of aggressive behavior, such as high histological grade and vascular invasion. More importantly, low expression of ZSCAN4 was found to be an independent negative prognostic factor for DSS and MeFS in patients with UTUC or UBUC. In line with the finding from our initial expression profiling analysis of UBUC transcriptome (GSE31684), ZSCAN4 was identified as a tumor suppressor in UC. Previous studies mainly focused on the mechanism of telomere elongation of ZSCAN4 in embryonic stem cells [20]. The functional role and prognostic significance of ZSCAN4 in cancer have never been elucidated. This was the first study that investigates the prognostic significance of ZSCAN4 in a well-defined cohort of cancer patients. Assessment of ZSCAN4 expression in patients with UC could provide information for risk stratification and aid in treating patients in a personalized manner.

ZSCAN4 is a newly identified embryonic stem cell marker and is highly expressed exclusively in late 2-cell embryonic stem cells [21]. ZSCAN4 was responsible for attenuating the DNA damage response, improving genomic stability and promoting telomere elongation during reprogramming [20, 22, 23]. ZSCAN4, in combination with the Yamanaka factors (Oct3/4, Sox2, Klf4, and c-Myc), significantly promoted the efficiency of induced pluripotent stem (iPS) cell generation. During iPS cell formation, ZSCAN4 reduced DNA double-strand break (DSB) signals, characterized by decreased total phosphorylated histone H2AX (γ-H2AX) level during reprogramming [22]. γ-H2AX is formed rapidly after DSBs, and critical lesions can cause genomic instability and tumorigenesis [24].

Mammalian telomeres are composed of repetitive TTAGGG sequences that are responsible for formation of the capping structures, which are bound by telomere-binding factors called shelterin [25, 26]. The shelterin complex consists of a six subunit complex, including directly binding proteins telomeric repeat-binding factor 1 (TRF1), telomeric repeat-binding factor 2 (TRF2), and protection of telomeres 1 (POT1) and their associated proteins repressor/activator protein 1 (RAP1), TPP1 (Adrenocortical dysplasia protein homolog), and TRF1-interacting nuclear factor 2 (TIN2) [27, 28]. Overexpression of ZSCAN4 could trigger rapid telomere extension and inhibit TRF2, POT1b and RAP1 and which, in turn, suppresses spontaneous telomere sister chromatid exchange [22]. In breast cancer cells (MCF7) and osteosarcoma cells (SaOS2), ZSCAN4 has been found to be directly bound to RAP1 in the nucleus, possibly regulating shelterin complex-controlled telomere elongation in both telomerase positive and alternative lengthening of telomere pathways [29]. Interestingly, in these two types of cancer cells, the protein expression of ZSCAN4 was also dependent on RAP1. However, as mentioned before, the mRNA transcript level of RAP1 could be repressed by ZSCAN4 in embryonic stem cells [22]. Although direct binding between ZSCAN4 and RAP1 was evident, definite functional interaction between ZSCAN4 and RAP1 remains obscure.

Though the role of ZSCAN4 in embryonic stem cells became increasingly clear in recent years, little is known with respect to the biological function of ZSCAN4 in cancer cells. The expression of ZSCAN4 has been demonstrated in a small proportion of cancer cells, including cervical cancer cells (HeLa), breast cancer cells (MCF7) and osteosarcoma cells (SaOS2 and U2OS) [29]. Additionally, in head and neck squamous cell carcinoma (HNSCC), ZSCAN4 played an important role in facilitating chromatin remodeling and activating cancer stem cell factor expression, including OCT3/4, NANOG, KLF4, and SOX2. Depletion of ZSCAN4 was found to have inhibitory effect on tumor growth in HNSCC [30]. Moreover, Zhang et al. found that ZSCAN4 expression is increased in DNA-damaged stromal cells that leads to a senescence-associated secretory phenotype (SASP), mediated by the ATM/TRAF6/TAK1/p65 signaling axis [31]. They also disclosed that targeting TAK1 in vivo increases chemosensitization and promotes tumor regression. These aforementioned findings suggested that ZSCAN4 have oncogenic role in some cancer types, other than UC. Currently, there is no data available regarding the expression and biological function of ZSCAN4 in UC cells. In cancer cells, telomere maintenance is an important mechanism to keep immortality. Accordingly, in terms of the known biological function of telomere elongation of ZSCAN4, ZSCAN4 expression in cancer cells may aid in telomere elongation, prevent cellular senescence and maintain normal karyotype for many cell divisions, and which, subsequently, result in cell immortalization [20]. In addition, during reprogramming in iPS cells, *ZSCAN4* has been found to indirectly downregulate p53, a key tumor suppressor [22]. However, more studies are needed to clarify mechanisms about the tumor suppressor role of ZSCAN4 in UC.

High mitotic activity has been associated with progression and recurrence of non-muscle-invasive bladder cancer and could be a useful prognostic marker beyond tumor grades [32]. Impressively, many genes co-downregulated with *ZSCAN4* were implicated in the mitotic cell cycle (Fig. 5A–C). Initially, as cells transit from interphase to mitosis, diverse events occur to prepare for chromosome separation, including chromosome condensation (GO 1,905,821, fold enrichment 58.29), nuclear envelope breakdown, spindle assembly (GO 0,007,057, fold enrichment 72.86), and segregation and movement of duplicated centrosomes to opposite poles of the cell [33]. Subsequently, the mitotic spindle attaches to and lines up chromosomes at its center, known as the metaphase plate [34]. The representative shape of metaphase spindle is featured by mirror symmetry of sister chromatids alongside this equator. Afterwards, during anaphase (GO 0,010,997, fold enrichment 32.38), the mitotic spindle elongates (GO 0,000,022, fold enrichment 48.57) and the central spindle (GO 0,097,149, fold enrichment 97.15) emerges in the middle of the spindle [35]. Despite the similar organization of spindle and central spindle, they assemble at different times during the cell cycle. The central spindle generates as cells exit mitosis and modulates cleavage furrow formation and completion of daughter cell separation. Accordingly, the association among the level of *ZSCAN4* mRNA, its co-downregulated genes and mitosis regulation, as well as their roles in the suppression of UC progression warrant further analysis.

## Conclusion

In this study, we firstly identified that ZSCAN4 acts as a tumor suppressor in UC. In patients with UTUC or UBUC, low expression of ZSCAN4 was significantly associated with some aggressive clinicopathological parameters. Moreover, low ZSCAN4 expression served as an adverse prognostic factor for disease-specific survival and metastasis-free survival.

## Supplementary Information

**Additional file 1: Table 1. **The top 200 *genes positively correlated with ZSCAN4. ***Table 2. **The top 200 *genes negatively correlated with ZSCAN4.*

## Data Availability

The data generated or analyzed in the current study were available from the corresponding author on reasonable request.

## Abbreviations

ZSCAN4: Zinc finger and SCAN domain containing 4
UC: Urothelial carcinoma
UBUC: Urinary bladder urothelial carcinoma
UTUC: Upper urinary tract urothelial carcinomas
TCGA: The Cancer Genome Atlas (TCGA)
DSS: Disease-specific survival (DSS)
MeFS: Metastasis-free survival
GO: Gene ontology
iPS: Induced pluripotent stem
DSB: Double-strand break
HNSCC: Head and neck squamous cell carcinoma
SASP: Senescence-associated secretory phenotype
