# Supplementary material for: Low expression of ZSCAN4 predicts unfavorable outcome in urothelial carcinoma of upper urinary tract and urinary bladder
Source: World J Surg Oncol. 2023 Feb 25;21:62. doi: 10.1186/s12957-023-02948-4 (PMC9960215; doi:10.1186/s12957-023-02948-4)
Supplement: Supplementary file 1 — Additional file 1: Table 1. The top 200 genes positively correlated with ZSCAN4. Table 2. The top 200 genes negatively correlated with ZSCAN4. [file 12957_2023_2948_MOESM1_ESM.docx]

**Supplementary Table 1. The top 200 genes positively correlated with ZSCAN4.**

| **Correlated Gene** | **Cytoband** | **Spearman's Correlation** | **p-Value** | **q-Value** |
| --- | --- | --- | --- | --- |
| **ZNF211** | 19q13.43 | 0.658 | 6.31E-52 | **1.26E-47** |
| **ZNF321P** | 19q13.41 | 0.548 | 3.12E-33 | **3.12E-29** |
| **ZNF816** | 19q13.41 | 0.53 | 7.23E-31 | **4.83E-27** |
| **ZNF440** | 19p13.2 | 0.524 | 4.14E-30 | **2.08E-26** |
| **CROT** | 7q21.12 | 0.523 | 5.88E-30 | **2.35E-26** |
| **ZNF320** | 19q13.41 | 0.516 | 4.47E-29 | **1.49E-25** |
| **CYP3A7** | 7q22.1 | 0.504 | 1.47E-27 | **4.19E-24** |
| **ANKRD65** | 1p36.33 | 0.502 | 2.39E-27 | **5.99E-24** |
| **CYP3A5** | 7q22.1 | 0.483 | 3.65E-25 | **7.58E-22** |
| **CTSH** | 15q25.1 | 0.483 | 3.85E-25 | **7.58E-22** |
| **CYP4B1** | 1p33 | 0.482 | 4.16E-25 | **7.58E-22** |
| **TMEM51-AS1** | 1p36.21 | 0.481 | 5.41E-25 | **8.39E-22** |
| **PRICKLE4** | 6p21.1 | 0.481 | 5.45E-25 | **8.39E-22** |
| **ZNF846** | 19p13.2 | 0.479 | 8.71E-25 | **1.25E-21** |
| **TEAD3** | 6p21.31 | 0.479 | 9.38E-25 | **1.25E-21** |
| **PLCH2** | 1p36.32 | 0.477 | 1.44E-24 | **1.80E-21** |
| **TP53TG1** | 7q21.12 | 0.476 | 2.22E-24 | **2.62E-21** |
| **ZNF626** | 19p12 | 0.47 | 9.20E-24 | **1.02E-20** |
| **SRC** | 20q11.23 | 0.469 | 1.28E-23 | **1.35E-20** |
| **ABAT** | 16p13.2 | 0.466 | 2.48E-23 | **2.48E-20** |
| **ZNF415** | 19q13.42 | 0.466 | 2.67E-23 | **2.52E-20** |
| **ZNF552** | 19q13.43 | 0.466 | 2.77E-23 | **2.52E-20** |
| **SSH3** | 11q13.2 | 0.46 | 1.03E-22 | **8.67E-20** |
| **ZNF737** | 19p12 | 0.46 | 1.04E-22 | **8.67E-20** |
| **BCAS1** | 20q13.2 | 0.459 | 1.39E-22 | **1.12E-19** |
| **TMEM63A** | 1q42.12 | 0.459 | 1.50E-22 | **1.12E-19** |
| **CAPS** | 19p13.3 | 0.457 | 2.15E-22 | **1.54E-19** |
| **VIPR1** | 3p22.1 | 0.456 | 2.89E-22 | **2.00E-19** |
| **CYP2J2** | 1p32.1 | 0.455 | 3.36E-22 | **2.17E-19** |
| **SLC44A3** | 1p21.3 | 0.455 | 3.36E-22 | **2.17E-19** |
| **ZNF83** | 19q13.41 | 0.455 | 3.77E-22 | **2.36E-19** |
| **CAB39L** | 13q14.2 | 0.454 | 4.23E-22 | **2.56E-19** |
| **IFT140** | 16p13.3 | 0.453 | 5.98E-22 | **3.49E-19** |
| **GGT6** | 17p13.2 | 0.453 | 6.10E-22 | **3.49E-19** |
| **HECTD2-AS1** | 10q23.32 | 0.45 | 1.02E-21 | **5.66E-19** |
| **PLA2G2F** | 1p36.12 | 0.45 | 1.17E-21 | **6.31E-19** |
| **LINC01341** | 1q44 | 0.45 | 1.20E-21 | **6.35E-19** |
| **ZNF506** | 19p13.11 | 0.448 | 1.73E-21 | **8.86E-19** |
| **LRMP** | 12p12.1 | 0.448 | 1.83E-21 | **9.16E-19** |
| **OVCH2** | 11p15.4 | 0.448 | 1.94E-21 | **9.50E-19** |
| **TMEM40** | 3p25.2 | 0.447 | 2.02E-21 | **9.65E-19** |
| **ZNF561** | 19p13.2 | 0.446 | 2.97E-21 | **1.38E-18** |
| **SYTL1** | 1p36.11 | 0.445 | 3.32E-21 | **1.51E-18** |
| **FER1L4** | 20q11.22 | 0.444 | 4.02E-21 | **1.75E-18** |
| **TMC7** | 16p12.3 | 0.444 | 4.17E-21 | **1.78E-18** |
| **MTSS2** | 16q22.1 | 0.444 | 4.57E-21 | **1.91E-18** |
| **ZNF432** | 19q13.41 | 0.442 | 6.77E-21 | **2.77E-18** |
| **ZNF223** | 19q13.31 | 0.44 | 1.18E-20 | **4.71E-18** |
| **ZNF137P** | 19q13.41 | 0.437 | 2.17E-20 | **8.36E-18** |
| **ZNF528** | 19q13.41 | 0.437 | 2.26E-20 | **8.45E-18** |
| **PLA2G4F** | 15q15.1 | 0.437 | 2.28E-20 | **8.45E-18** |
| **RGL2** | 6p21.32 | 0.436 | 2.51E-20 | **9.15E-18** |
| **PCP4L1** | 1q23.3 | 0.435 | 3.22E-20 | **1.15E-17** |
| **ZNF439** | 19p13.2 | 0.434 | 3.82E-20 | **1.34E-17** |
| **MAPK10** | 4q21.3 | 0.433 | 4.93E-20 | **1.67E-17** |
| **CMYA5** | 5q14.1 | 0.432 | 6.07E-20 | **1.99E-17** |
| **BAIAP3** | 16p13.3 | 0.431 | 7.20E-20 | **2.33E-17** |
| **TBX6** | 16p11.2 | 0.431 | 8.37E-20 | **2.66E-17** |
| **TMEM184A** | 7p22.3 | 0.43 | 1.02E-19 | **3.21E-17** |
| **SCCPDH** | 1q44 | 0.427 | 1.75E-19 | **5.38E-17** |
| **MAPK8IP3** | 16p13.3 | 0.427 | 1.81E-19 | **5.49E-17** |
| **VSIG2** | 11q24.2 | 0.426 | 2.06E-19 | **6.16E-17** |
| **AGAP11** | 10q23.2 | 0.426 | 2.48E-19 | **7.19E-17** |
| **DGKA** | 12q13.2 | 0.423 | 4.63E-19 | **1.27E-16** |
| **CAPNS2** | 16q12.2 | 0.421 | 6.89E-19 | **1.84E-16** |
| **ZNF665** | 19q13.42 | 0.419 | 9.62E-19 | **2.47E-16** |
| **PAQR7** | 1p36.11 | 0.419 | 1.03E-18 | **2.62E-16** |
| **SNCG** | 10q23.2 | 0.419 | 1.09E-18 | **2.72E-16** |
| **TMC4** | 19q13.42 | 0.418 | 1.22E-18 | **3.03E-16** |
| **CNGA1** | 4p12 | 0.418 | 1.30E-18 | **3.13E-16** |
| **JMJD7-PLA2G4B** | 15q15.1 | 0.416 | 1.77E-18 | **4.22E-16** |
| **METTL7A** | 12q13.12 | 0.415 | 2.15E-18 | **5.01E-16** |
| **ZNF222** | 19q13.31 | 0.415 | 2.29E-18 | **5.28E-16** |
| **S100P** | 4p16.1 | 0.415 | 2.32E-18 | **5.28E-16** |
| **UCA1** | 19p13.12 | 0.414 | 2.99E-18 | **6.69E-16** |
| **ZNF486** | 19p12 | 0.413 | 3.13E-18 | **6.81E-16** |
| **EDARADD** | 1q42.3-q43 | 0.413 | 3.41E-18 | **7.34E-16** |
| **CTSE** | 1q32.1 | 0.413 | 3.54E-18 | **7.54E-16** |
| **ZNF493** | 19p12 | 0.413 | 3.65E-18 | **7.69E-16** |
| **KRTAP5-9** | 11q13.4 | 0.411 | 4.63E-18 | **9.57E-16** |
| **OVGP1** | 1p13.2 | 0.411 | 5.16E-18 | **1.04E-15** |
| **FAM13A** | 4q22.1 | 0.41 | 5.76E-18 | **1.15E-15** |
| **ADAD2** | 16q24.1 | 0.41 | 5.78E-18 | **1.15E-15** |
| **CYP4Z2P** | 1p33 | 0.409 | 7.36E-18 | **1.42E-15** |
| **ZNF702P** | 19q13.41 | 0.409 | 7.68E-18 | **1.47E-15** |
| **SMIM5** | 17q25.1 | 0.409 | 8.21E-18 | **1.55E-15** |
| **ZNF69** | 19p13.2 | 0.408 | 8.44E-18 | **1.58E-15** |
| **ACER2** | 9p22.1 | 0.408 | 1.01E-17 | **1.88E-15** |
| **ZNF763** | 19p13.2 | 0.408 | 1.03E-17 | **1.89E-15** |
| **ZNF547** | 19q13.43 | 0.407 | 1.14E-17 | **2.08E-15** |
| **TOB1** | 17q21.33 | 0.407 | 1.18E-17 | **2.12E-15** |
| **ZNF600** | 19q13.41 | 0.407 | 1.18E-17 | **2.12E-15** |
| **PDXDC2P** | - | 0.407 | 1.22E-17 | **2.17E-15** |
| **PCMTD1** | 8q11.23 | 0.406 | 1.35E-17 | **2.36E-15** |
| **GRAMD2B** | 5q23.2 | 0.406 | 1.48E-17 | **2.51E-15** |
| **C19ORF18** | 19q13.43 | 0.405 | 1.72E-17 | **2.85E-15** |
| **ARHGAP23** | 17q12 | 0.405 | 1.77E-17 | **2.90E-15** |
| **SLC44A2** | 19p13.2 | 0.405 | 1.85E-17 | **3.01E-15** |
| **DENND2D** | 1p13.3-p13.2 | 0.404 | 1.88E-17 | **3.03E-15** |
| **PKIA** | 8q21.13 | 0.402 | 3.13E-17 | **4.94E-15** |
| **ZNF91** | 19p12 | 0.401 | 3.48E-17 | **5.45E-15** |
| **ZNF799** | 19p13.2 | 0.401 | 3.63E-17 | **5.59E-15** |
| **HMGCS2** | 1p12 | 0.401 | 3.97E-17 | **6.07E-15** |
| **RNF128** | Xq22.3 | 0.4 | 4.16E-17 | **6.28E-15** |
| **CAMK2G** | 10q22.2 | 0.4 | 4.23E-17 | **6.28E-15** |
| **PLEKHN1** | 1p36.33 | 0.4 | 4.24E-17 | **6.28E-15** |
| **SAMD10** | 20q13.33 | 0.399 | 5.47E-17 | **7.88E-15** |
| **PERM1** | 1p36.33 | 0.398 | 7.26E-17 | **1.04E-14** |
| **ZNF44** | 19p13.2 | 0.398 | 7.37E-17 | **1.05E-14** |
| **ZNF682** | 19p12 | 0.397 | 7.85E-17 | **1.10E-14** |
| **VAV3** | 1p13.3 | 0.397 | 8.24E-17 | **1.13E-14** |
| **PIK3C2B** | 1q32.1 | 0.397 | 8.26E-17 | **1.13E-14** |
| **ST3GAL5** | 2p11.2 | 0.396 | 9.23E-17 | **1.26E-14** |
| **PADI3** | 1p36.13 | 0.395 | 1.13E-16 | **1.52E-14** |
| **IL20RA** | 6q23.3 | 0.395 | 1.17E-16 | **1.56E-14** |
| **TBC1D3** | 17q12 | 0.395 | 1.19E-16 | **1.58E-14** |
| **SHROOM1** | 5q31.1 | 0.395 | 1.24E-16 | **1.63E-14** |
| **NADSYN1** | 11q13.4 | 0.395 | 1.30E-16 | **1.68E-14** |
| **ACSF2** | 17q21.33 | 0.394 | 1.33E-16 | **1.69E-14** |
| **CACNA1D** | 3p21.1 | 0.394 | 1.34E-16 | **1.69E-14** |
| **PTPRU** | 1p35.3 | 0.394 | 1.42E-16 | **1.78E-14** |
| **ZNF433** | 19p13.2 | 0.394 | 1.50E-16 | **1.87E-14** |
| **DHRS2** | 14q11.2 | 0.393 | 1.67E-16 | **2.06E-14** |
| **PSCA** | 8q24.3 | 0.393 | 1.68E-16 | **2.07E-14** |
| **KRTAP5-8** | 11q13.4 | 0.393 | 1.88E-16 | **2.29E-14** |
| **UGT2B28** | 4q13.2 | 0.392 | 1.98E-16 | **2.39E-14** |
| **SMIM6** | 17q25.1 | 0.392 | 2.15E-16 | **2.56E-14** |
| **ISYNA1** | 19p13.11 | 0.392 | 2.18E-16 | **2.58E-14** |
| **NDRG2** | 14q11.2 | 0.392 | 2.29E-16 | **2.69E-14** |
| **RGS12** | 4p16.3 | 0.391 | 2.40E-16 | **2.80E-14** |
| **ZNF429** | 19p12 | 0.391 | 2.45E-16 | **2.83E-14** |
| **ZNF177** | 19p13.2 | 0.391 | 2.47E-16 | **2.84E-14** |
| **KCNN4** | 19q13.31 | 0.391 | 2.58E-16 | **2.95E-14** |
| **ATP2C2** | 16q24.1 | 0.391 | 2.75E-16 | **3.11E-14** |
| **MPZL2** | 11q23.3 | 0.39 | 2.88E-16 | **3.24E-14** |
| **TRPV6** | 7q34 | 0.39 | 3.03E-16 | **3.39E-14** |
| **TBC1D3B** | 17q12 | 0.39 | 3.21E-16 | **3.57E-14** |
| **PLEKHH1** | 14q24.1 | 0.389 | 3.49E-16 | **3.84E-14** |
| **UPK2** | 11q23.3 | 0.389 | 3.59E-16 | **3.93E-14** |
| **ESRP2** | 16q22.1 | 0.389 | 3.88E-16 | **4.22E-14** |
| **ZNF823** | 19p13.2 | 0.388 | 4.22E-16 | **4.57E-14** |
| **WNT7B** | 22q13.31 | 0.388 | 4.92E-16 | **5.30E-14** |
| **CYP4F22** | 19p13.12 | 0.387 | 5.06E-16 | **5.41E-14** |
| **BTG2** | 1q32.1 | 0.387 | 5.53E-16 | **5.85E-14** |
| **TMEM51** | 1p36.21 | 0.386 | 6.00E-16 | **6.33E-14** |
| **CDC42BPG** | 11q13.1 | 0.386 | 6.06E-16 | **6.35E-14** |
| **SERINC4** | 15q15.3 | 0.386 | 6.18E-16 | **6.45E-14** |
| **TBC1D8** | 2q11.2 | 0.386 | 6.32E-16 | **6.52E-14** |
| **ALDH4A1** | 1p36.13 | 0.386 | 6.48E-16 | **6.65E-14** |
| **DDR1** | 6p21.33 | 0.386 | 6.73E-16 | **6.87E-14** |
| **UPK1A** | 19q13.12 | 0.385 | 7.59E-16 | **7.66E-14** |
| **BICDL2** | 16p13.3 | 0.385 | 7.80E-16 | **7.77E-14** |
| **TNFRSF21** | 6p12.3 | 0.385 | 7.91E-16 | **7.82E-14** |
| **LINC00663** | 19p13.11 | 0.385 | 7.94E-16 | **7.82E-14** |
| **ALDH1L1** | 3q21.3 | 0.385 | 8.50E-16 | **8.15E-14** |
| **PROM2** | 2q11.1 | 0.384 | 9.35E-16 | **8.88E-14** |
| **ZNF814** | 19q13.43 | 0.384 | 9.46E-16 | **8.94E-14** |
| **TSPAN6** | Xq22.1 | 0.384 | 9.84E-16 | **9.25E-14** |
| **RAPGEFL1** | 17q21.1 | 0.384 | 9.97E-16 | **9.33E-14** |
| **CAPN8** | 1q41 | 0.384 | 1.01E-15 | **9.39E-14** |
| **SLC23A2** | 20p13 | 0.383 | 1.16E-15 | **1.07E-13** |
| **NFAT5** | 16q22.1 | 0.383 | 1.19E-15 | **1.08E-13** |
| **ZNF443** | 19p13.2 | 0.382 | 1.33E-15 | **1.19E-13** |
| **KRT33A** | 17q21.2 | 0.382 | 1.44E-15 | **1.28E-13** |
| **CYP4F12** | 19p13.12 | 0.382 | 1.47E-15 | **1.31E-13** |
| **HAVCR1P1** | 19p12 | 0.381 | 1.52E-15 | **1.33E-13** |
| **BNIPL** | 1q21.3 | 0.381 | 1.53E-15 | **1.34E-13** |
| **SPERT** | 13q14.13 | 0.381 | 1.57E-15 | **1.36E-13** |
| **ARHGAP27** | 17q21.31 | 0.38 | 1.84E-15 | **1.56E-13** |
| **DUOX2** | 15q21.1 | 0.38 | 2.00E-15 | **1.67E-13** |
| **FAM183A** | 1p34.2 | 0.38 | 2.02E-15 | **1.68E-13** |
| **TBX3** | 12q24.21 | 0.38 | 2.05E-15 | **1.70E-13** |
| **BTBD16** | 10q26.13 | 0.379 | 2.17E-15 | **1.78E-13** |
| **SOX13** | 1q32.1 | 0.379 | 2.20E-15 | **1.80E-13** |
| **ABCA10** | 17q24.3 | 0.379 | 2.29E-15 | **1.86E-13** |
| **MIR22HG** | 17p13.3 | 0.379 | 2.34E-15 | **1.90E-13** |
| **SULT2A1** | 19q13.33 | 0.379 | 2.44E-15 | **1.97E-13** |
| **SNX31** | 8q22.3 | 0.379 | 2.60E-15 | **2.09E-13** |
| **CHAD** | 17q21.33 | 0.378 | 2.70E-15 | **2.16E-13** |
| **TTC23L** | 5p13.2 | 0.378 | 2.74E-15 | **2.19E-13** |
| **ZNF776** | 19q13.43 | 0.378 | 2.82E-15 | **2.24E-13** |
| **FAM214A** | 15q21.2-q21.3 | 0.377 | 3.38E-15 | **2.65E-13** |
| **SH3GLB2** | 9q34.11 | 0.377 | 3.38E-15 | **2.65E-13** |
| **PPFIBP2** | 11p15.4 | 0.376 | 4.05E-15 | **3.14E-13** |
| **DOK7** | 4p16.3 | 0.376 | 4.23E-15 | **3.26E-13** |
| **SYTL4** | Xq22.1 | 0.376 | 4.24E-15 | **3.26E-13** |
| **CYP4F29P** | 21q11.2 | 0.376 | 4.26E-15 | **3.26E-13** |
| **ZNF875** | 19q13.12 | 0.375 | 4.58E-15 | **3.46E-13** |
| **GTF2IRD2** | 7q11.23 | 0.375 | 4.64E-15 | **3.50E-13** |
| **PHGR1** | 15q15.1 | 0.375 | 4.68E-15 | **3.51E-13** |
| **HIP1R** | 12q24.31 | 0.375 | 4.87E-15 | **3.64E-13** |
| **ATF7IP2** | 16p13.2-p13.13 | 0.374 | 5.43E-15 | **4.00E-13** |
| **GCNT4** | 5q13.3 | 0.374 | 5.60E-15 | **4.11E-13** |
| **SH3YL1** | 2p25.3 | 0.374 | 5.82E-15 | **4.26E-13** |
| **IKZF2** | 2q34 | 0.373 | 6.46E-15 | **4.66E-13** |
| **TRIM62** | 1p35.1 | 0.373 | 6.49E-15 | **4.66E-13** |
| **CYP4Z1** | 1p33 | 0.373 | 7.48E-15 | **5.31E-13** |
| **PCGF3** | 4p16.3 | 0.372 | 7.84E-15 | **5.53E-13** |
| **CD46** | 1q32.2 | 0.372 | 8.10E-15 | **5.69E-13** |
| **HSD17B2** | 16q23.3 | 0.372 | 8.13E-15 | **5.69E-13** |

**Supplementary Table 2. The top 200 genes negatively correlated with ZSCAN4.**

| **Correlated Gene** | **Cytoband** | **Spearman's Correlation** | **p-Value** | **q-Value** |
| --- | --- | --- | --- | --- |
| **CMSS1** | 3q12.1 | -0.459 | 1.48E-22 | **1.12E-19** |
| **USP13** | 3q26.33 | -0.445 | 3.58E-21 | **1.59E-18** |
| **PRELID3A** | 18p11.21 | -0.437 | 1.90E-20 | **7.46E-18** |
| **ORC1** | 1p32.3 | -0.434 | 4.28E-20 | **1.48E-17** |
| **CDV3** | 3q22.1 | -0.432 | 5.83E-20 | **1.95E-17** |
| **MTHFD2** | 2p13.1 | -0.426 | 2.24E-19 | **6.58E-17** |
| **TEAD4** | 12p13.33 | -0.425 | 2.96E-19 | **8.46E-17** |
| **OGFRL1** | 6q13 | -0.424 | 3.27E-19 | **9.22E-17** |
| **POPDC3** | 6q21 | -0.424 | 3.54E-19 | **9.85E-17** |
| **KIF18A** | 11p14.1 | -0.421 | 6.45E-19 | **1.75E-16** |
| **FKBP5** | 6p21.31 | -0.42 | 8.07E-19 | **2.13E-16** |
| **TMEM131L** | 4q31.3 | -0.419 | 9.58E-19 | **2.47E-16** |
| **RELT** | 11q13.4 | -0.418 | 1.25E-18 | **3.05E-16** |
| **PARVB** | 22q13.31 | -0.416 | 2.01E-18 | **4.74E-16** |
| **RUVBL1** | 3q21.3 | -0.414 | 3.04E-18 | **6.69E-16** |
| **LMNB2** | 19p13.3 | -0.414 | 3.04E-18 | **6.69E-16** |
| **MYBL2** | 20q13.12 | -0.412 | 4.24E-18 | **8.85E-16** |
| **ANP32B** | 9q22.33 | -0.411 | 5.05E-18 | **1.03E-15** |
| **LRR1** | 14q21.3 | -0.41 | 6.01E-18 | **1.18E-15** |
| **SGTB** | 5q12.3 | -0.409 | 7.12E-18 | **1.38E-15** |
| **ADD2** | 2p13.3 | -0.407 | 1.25E-17 | **2.19E-15** |
| **SGO2** | 2q33.1 | -0.406 | 1.42E-17 | **2.45E-15** |
| **CDC25B** | 20p13 | -0.406 | 1.46E-17 | **2.49E-15** |
| **FOXM1** | 12p13.33 | -0.406 | 1.53E-17 | **2.57E-15** |
| **DENND1A** | 9q33.3 | -0.405 | 1.55E-17 | **2.58E-15** |
| **NEIL3** | 4q34.3 | -0.403 | 2.52E-17 | **4.04E-15** |
| **CKAP5** | 11p11.2 | -0.402 | 3.06E-17 | **4.86E-15** |
| **PRDX6** | 1q25.1 | -0.401 | 3.62E-17 | **5.59E-15** |
| **CEP55** | 10q23.33 | -0.4 | 4.23E-17 | **6.28E-15** |
| **MCM6** | 2q21.3 | -0.4 | 4.45E-17 | **6.55E-15** |
| **SSR3** | 3q25.31 | -0.4 | 4.96E-17 | **7.25E-15** |
| **KPNA2** | 17q24.2 | -0.399 | 5.35E-17 | **7.76E-15** |
| **DLGAP5** | 14q22.3 | -0.397 | 7.77E-17 | **1.10E-14** |
| **SNX10** | 7p15.2 | -0.397 | 8.07E-17 | **1.12E-14** |
| **CIP2A** | 3q13.13 | -0.396 | 1.07E-16 | **1.44E-14** |
| **SMC2** | 9q31.1 | -0.395 | 1.23E-16 | **1.63E-14** |
| **FMNL2** | 2q23.3 | -0.395 | 1.29E-16 | **1.68E-14** |
| **MELK** | 9p13.2 | -0.394 | 1.32E-16 | **1.69E-14** |
| **DCLRE1B** | 1p13.2 | -0.394 | 1.33E-16 | **1.69E-14** |
| **GAL** | 11q13.2 | -0.392 | 1.96E-16 | **2.38E-14** |
| **TPX2** | 20q11.21 | -0.392 | 2.01E-16 | **2.41E-14** |
| **NLN** | 5q12.3 | -0.392 | 2.25E-16 | **2.65E-14** |
| **CDCA5** | 11q13.1 | -0.391 | 2.68E-16 | **3.05E-14** |
| **PRAME** | 22q11.22 | -0.389 | 3.42E-16 | **3.79E-14** |
| **KIF4A** | Xq13.1 | -0.387 | 5.11E-16 | **5.44E-14** |
| **MAP7D3** | Xq26.3 | -0.386 | 6.25E-16 | **6.49E-14** |
| **CCNB2** | 15q22.2 | -0.386 | 6.81E-16 | **6.92E-14** |
| **FEN1** | 11q12.2 | -0.385 | 7.61E-16 | **7.66E-14** |
| **ME1** | 6q14.2 | -0.385 | 7.80E-16 | **7.77E-14** |
| **AIMP2** | 7p22.1 | -0.385 | 7.98E-16 | **7.82E-14** |
| **LDLRAD3** | 11p13 | -0.385 | 8.01E-16 | **7.82E-14** |
| **EIF5A2** | 3q26.2 | -0.385 | 8.15E-16 | **7.91E-14** |
| **GNB4** | 3q26.33 | -0.385 | 8.18E-16 | **7.91E-14** |
| **CENPA** | 2p23.3 | -0.385 | 8.33E-16 | **8.02E-14** |
| **C1ORF216** | 1p34.3 | -0.384 | 8.93E-16 | **8.52E-14** |
| **FXN** | 9q21.11 | -0.384 | 1.01E-15 | **9.39E-14** |
| **ASPM** | 1q31.3 | -0.383 | 1.06E-15 | **9.76E-14** |
| **PRXL2C** | 9q22.33 | -0.383 | 1.17E-15 | **1.07E-13** |
| **MT1G** | 16q13 | -0.382 | 1.29E-15 | **1.17E-13** |
| **CDK5RAP2** | 9q33.2 | -0.382 | 1.33E-15 | **1.19E-13** |
| **CDKN3** | 14q22.2 | -0.382 | 1.42E-15 | **1.27E-13** |
| **UAP1** | 1q23.3 | -0.382 | 1.50E-15 | **1.32E-13** |
| **KIF11** | 10q23.33 | -0.381 | 1.54E-15 | **1.35E-13** |
| **CENPO** | 2p23.3 | -0.381 | 1.68E-15 | **1.45E-13** |
| **POLD1** | 19q13.33 | -0.381 | 1.71E-15 | **1.47E-13** |
| **DEPDC1** | 1p31.3 | -0.38 | 1.82E-15 | **1.56E-13** |
| **PLK1** | 16p12.2 | -0.38 | 1.84E-15 | **1.56E-13** |
| **UCHL1** | 4p13 | -0.38 | 1.85E-15 | **1.57E-13** |
| **GPATCH4** | 1q22-q23.1 | -0.38 | 1.91E-15 | **1.61E-13** |
| **FANCC** | 9q22.32 | -0.38 | 1.94E-15 | **1.63E-13** |
| **MAD2L2** | 1p36.22 | -0.38 | 2.15E-15 | **1.77E-13** |
| **POLA2** | 11q13.1 | -0.377 | 3.15E-15 | **2.49E-13** |
| **RACGAP1** | 12q13.12 | -0.377 | 3.35E-15 | **2.64E-13** |
| **FSD1L** | 9q31.2 | -0.377 | 3.58E-15 | **2.79E-13** |
| **SLC16A1** | 1p13.2 | -0.376 | 4.11E-15 | **3.18E-13** |
| **MCM10** | 10p13 | -0.376 | 4.36E-15 | **3.32E-13** |
| **NCAPH** | 2q11.2 | -0.376 | 4.46E-15 | **3.39E-13** |
| **SPC25** | 2q24.3 | -0.375 | 5.13E-15 | **3.82E-13** |
| **QSOX2** | 9q34.3 | -0.375 | 5.23E-15 | **3.87E-13** |
| **ODC1** | 2p25.1 | -0.375 | 5.24E-15 | **3.87E-13** |
| **EXO1** | 1q43 | -0.374 | 5.89E-15 | **4.28E-13** |
| **YWHAH** | 22q12.3 | -0.374 | 5.89E-15 | **4.28E-13** |
| **POLD2** | 7p13 | -0.374 | 6.13E-15 | **4.43E-13** |
| **KATNAL1** | 13q12.3 | -0.373 | 6.92E-15 | **4.95E-13** |
| **CDC20** | 1p34.2 | -0.373 | 7.19E-15 | **5.12E-13** |
| **TARS** | 5p13.3 | -0.373 | 7.62E-15 | **5.39E-13** |
| **CENPL** | 1q25.1 | -0.372 | 8.44E-15 | **5.89E-13** |
| **CHAC2** | 2p16.2 | -0.372 | 8.56E-15 | **5.93E-13** |
| **PRC1** | 15q26.1 | -0.372 | 8.56E-15 | **5.93E-13** |
| **SACS** | 13q12.12 | -0.372 | 8.98E-15 | **6.16E-13** |
| **HASPIN** | 17p13.2 | -0.371 | 9.67E-15 | **6.57E-13** |
| **PCOLCE2** | 3q23 | -0.371 | 1.04E-14 | **6.96E-13** |
| **MSANTD3** | 9q31.1 | -0.37 | 1.12E-14 | **7.49E-13** |
| **PSMB5** | 14q11.2 | -0.37 | 1.22E-14 | **8.05E-13** |
| **HPSE** | 4q21.23 | -0.366 | 2.21E-14 | **1.41E-12** |
| **CGAS** | 6q13 | -0.366 | 2.29E-14 | **1.46E-12** |
| **B4GALNT1** | 12q13.3 | -0.366 | 2.31E-14 | **1.46E-12** |
| **RHEBL1** | 12q13.12 | -0.365 | 2.85E-14 | **1.76E-12** |
| **CENPW** | 6q22.32 | -0.365 | 2.97E-14 | **1.81E-12** |
| **SRSF12** | 6q15 | -0.364 | 3.33E-14 | **2.02E-12** |
| **KNSTRN** | 15q15.1 | -0.364 | 3.35E-14 | **2.02E-12** |
| **OXCT1** | 5p13.1 | -0.363 | 3.73E-14 | **2.22E-12** |
| **BUB1** | 2q13 | -0.363 | 3.74E-14 | **2.22E-12** |
| **TXNRD1** | 12q23.3 | -0.363 | 3.83E-14 | **2.27E-12** |
| **TRIP13** | 5p15.33 | -0.363 | 4.35E-14 | **2.55E-12** |
| **KIRREL2** | 19q13.12 | -0.362 | 4.41E-14 | **2.57E-12** |
| **KIF14** | 1q32.1 | -0.362 | 5.08E-14 | **2.89E-12** |
| **DIAPH3** | 13q21.2 | -0.361 | 5.37E-14 | **3.04E-12** |
| **ACAT1** | 11q22.3 | -0.361 | 5.63E-14 | **3.16E-12** |
| **IL12RB2** | 1p31.3 | -0.361 | 6.09E-14 | **3.39E-12** |
| **PRPF4** | 9q32 | -0.36 | 7.09E-14 | **3.89E-12** |
| **DDN** | 12q13.12 | -0.36 | 7.10E-14 | **3.89E-12** |
| **PRMT5** | 14q11.2 | -0.359 | 7.50E-14 | **4.08E-12** |
| **IMPA2** | 18p11.21 | -0.359 | 8.09E-14 | **4.33E-12** |
| **POLR1E** | 9p13.2 | -0.359 | 8.27E-14 | **4.42E-12** |
| **ADCY3** | 2p23.3 | -0.359 | 8.35E-14 | **4.43E-12** |
| **RRM1** | 11p15.4 | -0.358 | 9.60E-14 | **5.02E-12** |
| **MPP6** | 7p15.3 | -0.358 | 1.02E-13 | **5.29E-12** |
| **CCNB1** | 5q13.2 | -0.357 | 1.04E-13 | **5.37E-12** |
| **PSMD1** | 2q37.1 | -0.357 | 1.13E-13 | **5.78E-12** |
| **PRSS21** | 16p13.3 | -0.357 | 1.14E-13 | **5.78E-12** |
| **CHEK1** | 11q24.2 | -0.357 | 1.18E-13 | **5.97E-12** |
| **BMPR1B** | 4q22.3 | -0.357 | 1.19E-13 | **6.02E-12** |
| **TMEM38B** | 9q31.2 | -0.356 | 1.27E-13 | **6.36E-12** |
| **OSBPL6** | 2q31.2 | -0.356 | 1.35E-13 | **6.73E-12** |
| **KIF23** | 15q23 | -0.356 | 1.44E-13 | **7.11E-12** |
| **NUP155** | 5p13.2 | -0.355 | 1.48E-13 | **7.23E-12** |
| **IMPDH1** | 7q32.1 | -0.355 | 1.48E-13 | **7.24E-12** |
| **CCT4** | 2p15 | -0.355 | 1.55E-13 | **7.54E-12** |
| **MT1H** | 16q13 | -0.355 | 1.56E-13 | **7.55E-12** |
| **MSH6** | 2p16.3 | -0.355 | 1.61E-13 | **7.74E-12** |
| **SKA3** | 13q12.11 | -0.355 | 1.68E-13 | **8.05E-12** |
| **TRPS1** | 8q23.3 | -0.354 | 1.78E-13 | **8.43E-12** |
| **RFC4** | 3q27.3 | -0.354 | 2.01E-13 | **9.45E-12** |
| **FBXO5** | 6q25.2 | -0.353 | 2.04E-13 | **9.59E-12** |
| **NEK6** | 9q33.3 | -0.353 | 2.20E-13 | **1.03E-11** |
| **PSMD2** | 3q27.1 | -0.353 | 2.21E-13 | **1.03E-11** |
| **AGTPBP1** | 9q21.33 | -0.352 | 2.43E-13 | **1.10E-11** |
| **ASIC1** | 12q13.12 | -0.352 | 2.58E-13 | **1.17E-11** |
| **MCM2** | 3q21.3 | -0.351 | 2.87E-13 | **1.28E-11** |
| **PSMC3** | 11p11.2 | -0.351 | 2.91E-13 | **1.29E-11** |
| **HAT1** | 2q31.1 | -0.35 | 3.38E-13 | **1.49E-11** |
| **GMPS** | 3q25.31 | -0.35 | 3.39E-13 | **1.49E-11** |
| **MRPL37** | 1p32.3 | -0.35 | 3.48E-13 | **1.52E-11** |
| **MKI67** | 10q26.2 | -0.35 | 3.51E-13 | **1.53E-11** |
| **SHCBP1** | 16q11.2 | -0.35 | 3.60E-13 | **1.56E-11** |
| **CDCA2** | 8p21.2 | -0.35 | 3.63E-13 | **1.57E-11** |
| **SUPT16H** | 14q11.2 | -0.35 | 3.74E-13 | **1.61E-11** |
| **GPRIN1** | 5q35.2 | -0.35 | 3.77E-13 | **1.62E-11** |
| **PAQR4** | 16p13.3 | -0.35 | 3.79E-13 | **1.62E-11** |
| **LRP8** | 1p32.3 | -0.35 | 3.85E-13 | **1.64E-11** |
| **DYNC1LI1** | 3p22.3 | -0.349 | 3.93E-13 | **1.67E-11** |
| **EIF2B3** | 1p34.1 | -0.349 | 3.96E-13 | **1.68E-11** |
| **PIK3AP1** | 10q24.1 | -0.349 | 4.23E-13 | **1.79E-11** |
| **DBF4** | 7q21.12 | -0.349 | 4.41E-13 | **1.86E-11** |
| **IPO4** | 14q12 | -0.349 | 4.49E-13 | **1.89E-11** |
| **ECT2** | 3q26.31 | -0.349 | 4.54E-13 | **1.90E-11** |
| **RNASEH1** | 2p25.3 | -0.348 | 5.00E-13 | **2.06E-11** |
| **MAMLD1** | Xq28 | -0.347 | 5.40E-13 | **2.21E-11** |
| **NCAPG** | 4p15.31 | -0.347 | 5.64E-13 | **2.31E-11** |
| **KCNG3** | 2p21 | -0.347 | 5.66E-13 | **2.31E-11** |
| **PHF19** | 9q33.2 | -0.347 | 5.96E-13 | **2.40E-11** |
| **GEMIN2** | 14q21.1 | -0.347 | 5.97E-13 | **2.40E-11** |
| **SLC39A14** | 8p21.3 | -0.347 | 6.19E-13 | **2.49E-11** |
| **TUBB6** | 18p11.21 | -0.347 | 6.20E-13 | **2.49E-11** |
| **NDC80** | 18p11.32 | -0.347 | 6.21E-13 | **2.49E-11** |
| **GPR156** | 3q13.33 | -0.347 | 6.23E-13 | **2.49E-11** |
| **TUBA1B** | 12q13.12 | -0.347 | 6.30E-13 | **2.51E-11** |
| **IRAK1** | Xq28 | -0.347 | 6.33E-13 | **2.52E-11** |
| **WDR5** | 9q34.2 | -0.346 | 6.52E-13 | **2.58E-11** |
| **LRFN4** | 11q13.2 | -0.346 | 6.71E-13 | **2.64E-11** |
| **BIRC5** | 17q25.3 | -0.346 | 6.96E-13 | **2.74E-11** |
| **NCL** | 2q37.1 | -0.346 | 7.08E-13 | **2.77E-11** |
| **CAD** | 2p23.3 | -0.346 | 7.12E-13 | **2.79E-11** |
| **SCML2** | Xp22.13 | -0.346 | 7.15E-13 | **2.79E-11** |
| **PES1** | 22q12.2 | -0.345 | 8.20E-13 | **3.16E-11** |
| **STRAP** | 12p12.3 | -0.345 | 8.28E-13 | **3.18E-11** |
| **CCNA2** | 4q27 | -0.345 | 8.30E-13 | **3.19E-11** |
| **WDHD1** | 14q22.2-q22.3 | -0.345 | 8.57E-13 | **3.28E-11** |
| **ARHGAP11A** | 15q13.3 | -0.344 | 9.59E-13 | **3.64E-11** |
| **CEP128** | 14q31.1 | -0.344 | 9.66E-13 | **3.66E-11** |
| **EIF3B** | 7p22.3 | -0.344 | 9.94E-13 | **3.74E-11** |
| **MAP3K20** | 2q31.1 | -0.344 | 1.01E-12 | **3.78E-11** |
| **HSPD1** | 2q33.1 | -0.344 | 1.02E-12 | **3.80E-11** |
| **GPR161** | 1q24.2 | -0.343 | 1.04E-12 | **3.88E-11** |
| **KIF2C** | 1p34.1 | -0.343 | 1.04E-12 | **3.88E-11** |
| **KATNA1** | 6q25.1 | -0.343 | 1.09E-12 | **4.02E-11** |
| **BMP2K** | 4q21.21 | -0.343 | 1.16E-12 | **4.27E-11** |
| **COQ2** | 4q21.22-q21.23 | -0.342 | 1.22E-12 | **4.45E-11** |
| **YKT6** | 7p13 | -0.342 | 1.39E-12 | **5.02E-11** |
| **WDR76** | 15q15.3 | -0.341 | 1.50E-12 | **5.39E-11** |
| **ACTL8** | 1p36.13 | -0.341 | 1.54E-12 | **5.52E-11** |
| **SRPRB** | 3q22.1 | -0.341 | 1.62E-12 | **5.76E-11** |
| **CDC45** | 22q11.21 | -0.34 | 1.67E-12 | **5.94E-11** |
| **SLC6A15** | 12q21.31 | -0.34 | 1.75E-12 | **6.18E-11** |
| **DTL** | 1q32.3 | -0.34 | 1.88E-12 | **6.62E-11** |
| **MYO7A** | 11q13.5 | -0.34 | 1.93E-12 | **6.77E-11** |
| **STIP1** | 11q13.1 | -0.339 | 1.96E-12 | **6.85E-11** |
| **SHOX2** | 3q25.32 | -0.339 | 2.05E-12 | **7.15E-11** |
| **HJURP** | 2q37.1 | -0.339 | 2.12E-12 | **7.36E-11** |
